# Heme Oxygenase-1 Reduces Sepsis-Induced Endoplasmic Reticulum Stress and Acute Lung Injury

**DOI:** 10.1155/2018/9413876

**Published:** 2018-06-14

**Authors:** Xiaozhen Chen, Yinglin Wang, Xiang Xie, Hongfei Chen, Qiqi Zhu, Zhidong Ge, Hua Wei, Jingshong Deng, Zhengyuan Xia, Qingquan Lian

**Affiliations:** ^1^Department of Anesthesiology, The Second Affiliated Hospital and Yuying Children's Hospital, Wenzhou Medical University, Wenzhou, Zhejiang, China; ^2^Department of Anesthesiology, Shanghai East Hospital, Tongji University Hospital School of Medicine, Shanghai, China; ^3^Department of Anesthesiology, Gaozhou People's Hospital and Gaozhou Hospital of Guangdong Medical University, Gaozhou, Guangdong, China; ^4^Department of Anesthesiology, The University of Hong Kong, Hong Kong

## Abstract

**Background:**

Sepsis leads to severe acute lung injury/acute respiratory distress syndrome (ALI/ARDS) that is associated with enhanced endoplasmic reticulum (ER) stress. Heme oxygenase-1 (HO-1), an ER-anchored protein, exerts antioxidant and protective functions under ALI. However, the role of HO-1 activation in the development of endoplasmic reticulum (ER) stress during sepsis remains unknown.

**Methods:**

Cecal ligation and puncture (CLP) model was created to induce septic ALI. Lung tissue ER stress was measured 18 hours after CLP. The effects of HO-1 on ER stress during septic ALI were investigated *in vivo* using HO-1 agonist hemin and antagonist ZnPP.

**Results:**

Compared with the sham group, ER stress in septic lung increased significantly 18 hours after CLP, which was significantly reduced by pretreatment with the ER inhibitor 4-phenylbutyrate (4-PBA). The lung injury score and the lung wet to dry (W/D) ratio in lungs were significantly reduced in septic rats after ER stress inhibition. Similarly, lung ER stress-related genes' (PERK, eIF2-*α*, ATF4, and CHOP) levels were attenuated after ER stress inhibition. Furthermore, HO-1 activation by hemin reduced p-PERK, p-eIF2-*α*, ATF4, and CHOP protein expression and oxidative stress and lung cell apoptosis. Additionally, HO-1 antagonist could aggregate the ER stress-related ALI.

**Conclusions:**

ER stress was activated during CLP-induced ALI, which may represent a mechanism by which CLP induces ALI. HO-1 activation could inhibit CLP-induced lung ER stress and attenuate CLP-induced ALI.

## 1. Introduction

Sepsis and septic shock, caused by microorganisms such as bacteria, viruses, or parasites, are severe systemic inflammatory response syndromes in critically ill patients with severe trauma, burns, hemorrhage, and so on in the intensive care units (ICUs) [1–3]. Sepsis initiates overresponse of host defense which results in exacerbated hemodynamic instability, abnormal platelet, disseminated intravascular coagulation (DIC), and even multiorgan dysfunction syndrome (MODS) [4–7]. In ICUs, the deterioration of sepsis contributes to high mortality (30%–50%) [8] and results in an estimated 5.3 million deaths worldwide every year [3, 9]. Among the varieties of complications and MODS induced by sepsis, acute lung injury (ALI) and acute respiratory distress syndrome (ARDS) are frequent due to pulmonary susceptibility [10], which associated with an increased risk of in-hospital mortality in ICUs [11].

The sepsis-associated ALI leads to refractory hypoxemia and respiratory distress clinically, and the pathological characteristics manifest as alveolar epithelial cell and capillary endothelial cell damage, inflammatory cell infiltration, and pulmonary interstitial congestion and edema. Unfortunately, there has been neither effective preventive strategies nor suitable therapeutic options existed for the treatment or prevention of ALI due to the complications of etiology and pathogenesis in ALI. Researches regarding the pathogenesis process of ALI have been concentrated on uncontrolled inflammation activation, alveolar epithelial cell apoptosis, excessive oxidative stress, coagulation dysfunction, and so on [12, 13]. However, the precise mechanisms are still poorly understood, limiting the discovery of effective treatments.

The endoplasmic reticulum (ER) is an intracellular organelle where the protein molecule folding, transportation, or modification takes place and also a place for calcium storage, lipid synthesis, and carbohydrate metabolism [13, 14]. Accumulated evidences observed that the homeostasis of ER alters under certain pathological conditions, such as sepsis, trauma, ischemia, and viral infection, leading to the accumulation of misfolded or unfolded proteins and ER stress [15–18]. In recent years, some researches have revealed an interaction between ER stress and sepsis [19]. Ma et al. demonstrated that ER stress contributed to abnormal lymphocyte apoptosis during sepsis in mice [20]. Zhang et al. reported the upregulation of GRP94, CHOP (ER stress components) in myocardial depression of septic rats, and ER stress inhibition protected the myocardium [18]. Moreover, ER stress has been shown to be attributable to sepsis-induced pulmonary inflammation through NF-*κ*B/HIF-1*α* signaling pathway modulation [16]. These evidences suggest that ER stress may be a novel target in clinical therapy of sepsis and its complications.

Heme oxygenase (HO)-1 is an essential enzyme in heme catabolism physiologically, cleaving heme to biliverdin [21]. HO-1 catabolizes free heme, produces carbon monoxide (CO), and possesses anti-inflammatory properties through upregulation of interleukin 10 (IL-10) [22]. It has been reported that HO-1 suppresses oxidative stress in sepsis-induced ALI/ARDS, and the possible mechanism could be associated with the activation of PI3K/Akt pathway [23] or Nrf2 signaling pathway [24, 25]. Furthermore, HO-1 has been reported to prevent ER stress-mediated hepatic [26], endothelial [21] cell apoptosis or myocardial ischemia-reperfusion injury [27] in diabetic animals. However, the relationship between HO-1 and ER stress during sepsis-associated ALI is unknown. Therefore, the current experiment was designed to investigate the effects of HO-1 on ER stress in sepsis-associated ALI and explore the potential mechanisms.

## 2. Methods and Materials

### 2.1. Experiments Design

Following the approval by Institutional Animal Research Committee, specific pathogen-free (SPF) male Sprague-Dawley rats weighing 180–220 g were obtained and housed for 7 days after arrival in the animal facility before performing the experiments. Rats were fed with food and water ad libitum. Cecal ligation and puncture (CLP) model was created according to the classical method described previously [28]. Three parts were involved in the current experiments. In the first part of the experiment that was aimed at establishing the model and at observing status of ER stress and HO-1 expression, two groups (sham and CLP, *n* = 6 per group) of animals were studied. In the second part, rats receiving the ER stress inhibitor 4-phenylbutyrate (4-PBA) (40 mg/kg) given intraperitoneally 30 minutes (min) before operation underwent celiotomy with CLP and compared with rats which received CLP without 4-PBA pretreatment and with sham operated group (sham, CLP, CLP + PBA, *n* = 6 per group) were studied. Finally, in the third part, rats were intraperitoneally injected with saline, hemin (30 mg/kg) 24 h before operation with or without the HO-1 inhibitor ZnPP (20 mg/kg) given 12 h after hemin administration, then received celiotomy with or without CLP surgery. Three groups (CLP, CLP + hemin, and CLP + hemin + ZnPP, *n* = 6 per group) were studied. All animals were anaesthetized with isoflurane inhalation and terminated by high concentration of carbon dioxide at 18 h after CLP. The lung tissue was then quickly removed, and left lobe was snap frozen in liquid nitrogen and stored at −80°C until analyzed. The right upper lobe was collected for histological assay and the right middle lobe for measurement of lung water.

### 2.2. Pathological Assessment

The lung tissues were paraffined and sectioned at about 5 *μ*m thick and stained with hematoxylin and eosin (H&E) as described [29]. Lung injury was evaluated and scored by two pathologists blinded to the experimental design using a recently criterion [30] in which lung damage is evaluated on a two-point scale with the score ranging from 0 to 1.

### 2.3. Water Content Assay

The right middle lobe was used for measurement of lung water content. Lung lobes were weighed before (wet weight) and after (dry weight) drying for 24 h in an 80°C oven. The water content of the lung was calculated as: lung water content % = (wet weight − dry weight)/wet weight × 100.

### 2.4. MDA Assay

Lung tissues were prepared as 10% tissue homogenates and centrifuged at 3000 rpm for 10 min at 4°C. The supernatant was collected for further analysis. The MDA content was detected according to the instructions of the MDA kit (Nanjing Jiancheng Bioengineering Institute, Nanjing, China).

### 2.5. Real-Time PCR Assay

Total RNAs were extracted from the left lobe of the lung of rats in various groups by using the RNeasy Mini Kit (Qiagen, Hilden, Germany). DNase I-treated total RNA (3 *μ*g) was reverse transcribed with oligo-dT and SuperScript II reverse transcriptase for RNA kinetic analysis, which was performed according to the manufacturer's instructions (Invitrogen, Carlsbad, CA). Real-time PCR was carried out by detecting the change in fluorescence in real time of SYBR Green dye (Qiagen) by using the real-time PCR kits (Applied Biosystems, Foster, CA). PCR primer pairs were as following: ATF4 sense: 5′-ACCAGTCGGGTTTGGGGGCT-3′; ATF4 antisense: 5′-TTCCGAGGAGCCCGCCTTGT-3′; eIF2-*α* sense: 5′-GCTGCGAGTCAGTAAT GGGTATAA-3′; eIF2-*α* antisense: 5′-CTGCCAGGAAACTTGCCACA-3′; CHOP sense: 5′-AGATGAAATTGGGGGCACCTATATC-3′; CHOP antisense: 5′-AGC ATGCACTGGAGATTACTGCT-3′; PERK sense: 5′-TCCCTCCACCTCCATGTCA-3′; PERK antisense: 5′-CTTCCAGGCGAAGCGTAAT-3′; GAPDH sense: 5′-ACCACAGTCCATGCCATCAC-3′; GAPDH antisense: 5′-TCCACCACCCTG TTGCTGTA-3′.

### 2.6. Immunohistochemistry of p-PERK

Lung tissues were fixed in 4% formalin and embedded in paraffin as previously described [31]. 5 *μ*m lung paraffin sections were dewaxed, hydrated, and then incubated with anti-p-PERK antibody (diluted 1 : 200; Cell Signaling Technology, USA) at 4°C overnight. After biotin-labeled secondary antibody was added to the slides, slides were stained with 3,3′-diaminobenzidine (DAB) and counterstained with hematoxylin. Finally, the stained slides were observed by using a digital camera under microscope (Leica, DMLB2, Germany).

### 2.7. TUNEL-Positive Nucleus

Paraffin-embedded lung tissues were processed for immunochemistry. Apoptotic cells were detected by TdT-mediated dUTP nick end labeling (TUNEL) method [32]. The TUNEL assay was carried out according to the manufacturer's instructions (Roche Applied Sciences, Shanghai, China).

### 2.8. Western Blot Assay

Total proteins from lung tissues were extracted, and protein concentrations were determined by the bicinchoninic acid (BCA) protein assay (Nanjing KeyGen Biotech. Co. Ltd., China). Protein extracts at equal amount were separated on sodium dodecyl sulfate- (SDS-) polyacrylamide gels and subsequently transferred onto a polyvinylidene fluoride (PVDF) membrane. After blocked with nonfat milk (5%) in Tris-buffered saline (TBS) at room temperature for one hour, the membranes were then incubated at 4°C overnight with rabbit monoclonal anti-p-PERK (1 : 1000; Cell Signaling Technology, USA), rabbit monoclonal anti-p-eIF2-*α* (1 : 1000, Cell Signaling Technology), rabbit monoclonal anti-ATF4 (1 : 1000, Cell Signaling Technology), rabbit monoclonal anti-CHOP (1 : 1000, Cell Signaling Technology), rabbit monoclonal anti-Bcl-2 (1 : 1000, Cell Signaling Technology), rabbit monoclonal anti-Bax (1 : 1000, Cell Signaling Technology) or rabbit polyclonal anti-GAPDH (1 : 2000, Santa Cruz Biotechnology), and then with horse radish peroxidase-conjugated goat antirabbit IgG antibody (1 : 2000, Cell Signaling Technology). Optical density values of the Western blot bands were normalized to those of GAPDH.

### 2.9. Statistical Assay

All data are presented as mean ± standard error of the mean (SEM) from three independent experiments. Data were analyzed using one-way analysis of variance (ANOVA) followed by Tukey's post hoc test in SPSS13.0 (Chicago, IL, USA). *P* value < 0.05 was considered to be significant between groups.

## 3. Results

### 3.1. Pulmonary Injury Occurred during Sepsis with Concomitant Activation of Lung Tissue Endoplasmic Reticulum

**Figure 1:**
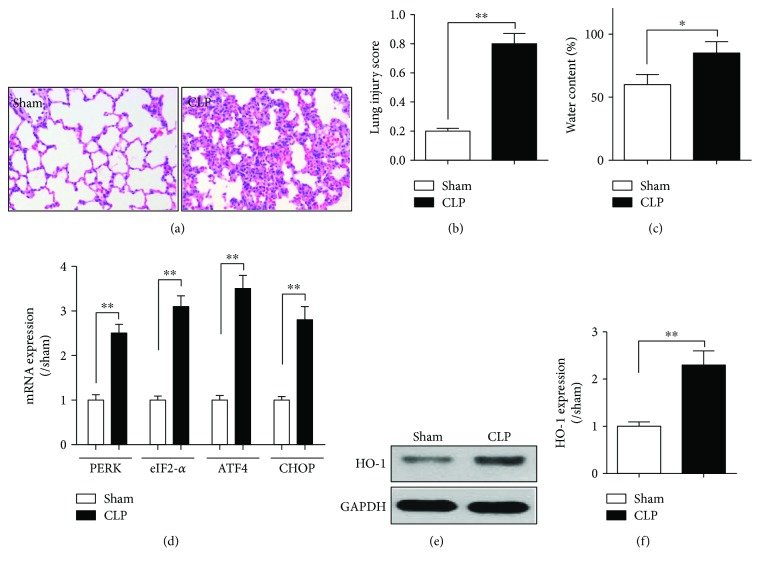
Pulmonary endoplasmic reticulum stress was activated during sepsis. Sprague-Dawley rats received cecal ligation and puncture (CLP) surgery or only laparotomy without CLP surgery (sham group). (a) Lung pathology was detected by using hematoxylin and eosin (H&E) staining. (b-c) Levels of the lung injury score and water content in lung tissues. After treatment with CLP for 18 h, lung injury score (b) evaluation was due to the pathology and levels of the water content (c) in lung tissues were analyzed by using W/D method. (d) Levels of endoplasmic reticulum stress-related genes, that is, PERK, eIF2-*α*, ATF4, and CHOP, were detected using real-time (RT) PCR method. (e) Level of HO-1 was detected by Western blot method, and gray analysis was performed according to the bands. PERK, double-stranded RNA-dependent protein kinase- (PKR-) like ER kinase; eIF2-*α*, eukaryotic initiation factor 2-*α*; ATF4, activating transcription factor 4; CHOP, C/EBP homologous protein. Data are shown as mean ± SEM (*n* = 6). ^∗^*P* < 0.05 and ^∗∗^*P* < 0.01; one-way ANOVA Tukey's post hoc test.

As shown in Figure 1(a), compared to the sham group, severe alveolar collapse, interstitial edema, and alveolar and mesenchymal hemorrhage were presented in the CLP group. Levels of lung injury scores and water content in lung tissues were evaluated in the CLP group as determined 18 h after CLP. As shown in Figures 1(b) and 1(c), levels of lung injury scores and water content in lung tissues increased significantly in the CLP group compared with that of the sham group (all *P* < 0.05). Levels of endoplasmic reticulum (ER) stress-related genes, that is, PERK, eIF2-*α*, ATF4, and CHOP in the lung tissue, were analyzed by real-time PCR at 18 h after CLP. As shown in Figure 1(d), levels of lung PERK, eIF2-*α*, ATF4, and CHOP mRNA expression in the CLP group were significantly higher than those in the sham group (*P* < 0.01), while lung tissue HO-1 protein expression in the CLP group was higher than that in the sham group (*P* < 0.01) (Figures 1(e) and 1(f)).

### 3.2. Inhibition of Endoplasmic Reticulum Stress Reduced Lung Injury in Sepsis Animals

**Figure 2:**
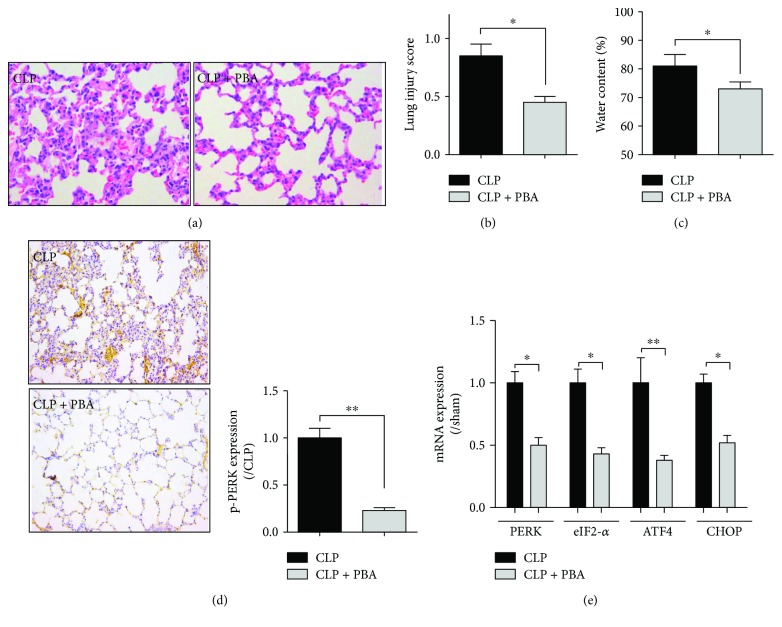
Inhibition of endoplasmic reticulum stress could reduce lung injury in sepsis animals. Sprague-Dawley rats were injected intraperitoneally with 4-PBA (40 mg/kg) 30 min before CLP, and lung samples were collected 18 h following CLP. (a) Lung pathology was detected by using hematoxylin and eosin (H&E) staining. (b-c) Levels of lung injury score and water content in lung tissues. Lung injury score (b) evaluation was due to the pathology, and levels of the water content (c) in lung tissues were analyzed by using W/D method. (d) The expression of p-PERK protein was assayed by immunohistochemical technique. (e) Levels of endoplasmic reticulum stress-related genes, that is, PERK, eIF2-*α*, ATF4, and CHOP, were detected using real-time (RT) PCR method. PBA, 4-PBA. Data are shown as mean ± SEM (*n* = 6). ^∗^*P* < 0.05 and ^∗∗^*P* < 0.01; one-way ANOVA Tukey's post hoc test.

To confirm whether ER stress contributes to the pathogenesis of septic ALI, ER stress inhibitor 4-PBA was applied in the *in vivo* experiments. *In vivo* results showed that 4-PBA significantly attenuated lung CLP-induced pathological alternations (Figure 2(a)) and reduced lung injury score (Figure 2(b)) and pulmonary edema (Figure 2(c)). To detect which of the ER stress pathway took part in the protective progress of 4-PBA, p-PERK (protein kinase RNA- (PKR-) like ER kinase) protein expression was measured by using immunohistochemical technique, and results showed that p-PERK protein expression was significantly suppressed after 4-PBA treatment (Figure 2(d)). Furthermore, ER-related genes including PERK, eIF2-*α*, ATF4, and CHOP were also assayed by RT-PCR. As shown in Figure 2(e), mRNA levels of PERK, eIF2-*α*, ATF4, and CHOP in lung were all suppressed by ER stress inhibitor 4-PBA. These results indicating ER stress may play an important role in septic ALI, and inhibition of ER stress may effectively improve lung function.

### 3.3. HO-1 Activation Could Inhibit Endoplasmic Reticulum Stress and Reduced ER Stress-Related Lung Injury during Sepsis

**Figure 3:**
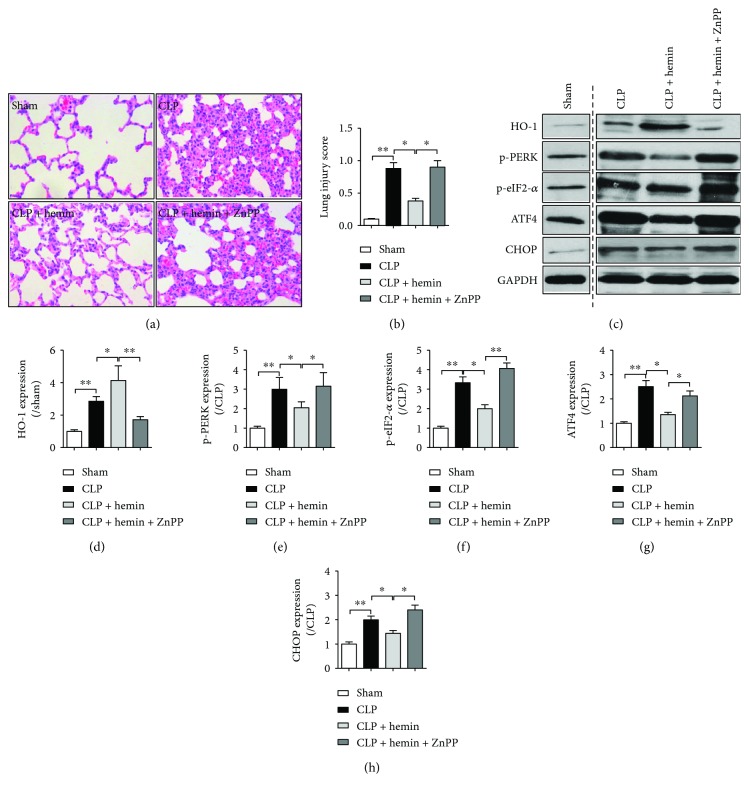
HO-1 activation could inhibit endoplasmic reticulum stress and reduced ER stress-related lung injury. Sprague-Dawley rats were intraperitoneally injected with saline, hemin (30 mg/kg) 24 h before operation with or without ZnPP (20 mg/kg) 12 h after hemin was given, then received celioectomy with or without CLP surgery. (a) Lung pathology was detected by using hematoxylin and eosin (H&E) staining. (b) Levels of lung injury score. Lung injury score evaluations were due to the pathology in lung H&E staining. (c–h) Levels of endoplasmic reticulum stress-related proteins, that is, HO-1, p-PERK, p-eIF2-*α*, ATF4, and CHOP, were detected using Western blot method, and gray analysis were performed according to the bands. p-PERK, phosphorylated PERK; p-eIF2-*α*, phosphorylated eIF2-*α*. Data are shown as mean ± SEM (*n* = 6). ^∗^*P* < 0.05 and ^∗∗^*P* < 0.01; one-way ANOVA Tukey's post hoc test.

To further confirm that whether activated HO-1 could affect lung ER stress during sepsis, HO-1 agonist hemin was used in the *in vivo* experiments. As shown in Figure 3(a), hemin pretreatment improved the construction of lung and reduced the lung injury score (Figure 3(b)). HO-1 protein expression was significantly increased by hemin pretreatment. Also, hemin pretreatment decreased the ER stress by downregulating ER-related proteins p-PERK, p-eIF2-*α*, ATF4, and CHOP expressions (Figures 3(c) and 3(e)–3(h)). Moreover, as shown in Figure 3, HO-1 antagonist ZnPP led to the suppression of HO-1 protein expression and subsequently aggregated septic ALI and cancelled hemin-induced protective effects. Compared with the group CLP + hemin, the ER stress-related proteins p-PERK, p-eIF2-*α*, ATF4, and CHOP expressions were greatly elevated after ZnPP pretreatment.

### 3.4. The Protective Effects of HO-1 Activation Were Associated with Lung Cell Apoptosis Reduction

**Figure 4:**
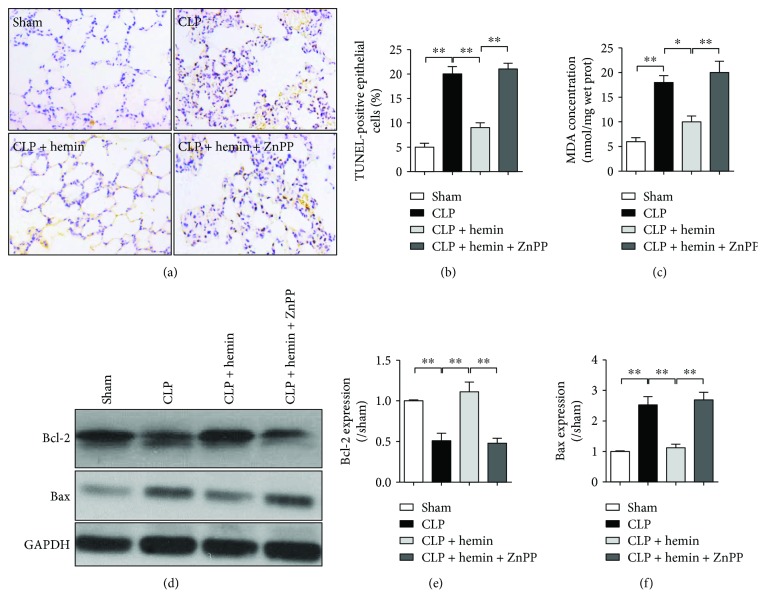
The protective effects of HO-1 activation and inhibition of ER stress were associated with lung cell apoptosis reduction. Sprague-Dawley rats were intraperitoneally injected with saline, hemin (30 mg/kg) 24 h before operation with or without ZnPP (20 mg/kg) 12 h after hemin was given, then received celioectomy with or without CLP surgery. Lung cell apoptosis was detected by using terminal deoxynucleotidyl transferase-mediated nick end labeling (TUNEL) method (a), and TUNEL-positive lung cells were counted (b). (c) Level of MDA concentration in lung tissue. (d–f) Levels of Bcl-2 and Bax were detected using Western blot method, and gray analysis were performed according to the bands. Data are shown as mean ± SEM (*n* = 6). ^∗^*P* < 0.05 and ^∗∗^*P* < 0.01; one-way ANOVA Tukey's post hoc test.

Given that cell apoptosis has been shown to play an important role in sepsis-induced ALI [33], we evaluated whether or not HO-1 activation protected lung from damage by reduced apoptosis during sepsis. Compared to the sham group, lung cell apoptosis was significantly increased 18 h after CLP, which indicated that lung cell apoptosis may be the important factor leading to lung dysfunction. As shown in Figures 4(a) and 4(b), HO-1 activation by hemin pretreatment could significantly attenuate lung apoptosis, and ZnPP eliminated the antiapoptotic effect of hemin. Also, we detected the MDA level which reflected lung lipid peroxidation and found that hemin treatment could effectively reduce the lung MDA level (Figure 4(c)). Furthermore, protein expressions of the antiapoptotic Bcl-2 and proapoptotic Bax 18 h after CLP were also detected. As shown in Figures 4(d)–4(f), compared with sham group, the Bcl-2 level was significantly decreased and Bax increased in the CLP group. Hemin pretreatment attenuated CLP-induced decrease of the Bcl-2 protein level and suppressed Bax. ZnPP reversed hemin-induced alterations of Bcl-2 and Bax expressions.

## 4. Discussion

There is convincing evidence indicating the role of ER stress in sepsis-associated ALI pathophysiology [16, 34, 35]. HO-1 has been reported to exert pulmonary protective effects in sepsis. It acts as a potent anti-inflammatory and antioxidant agent through its products carbon monoxide (CO) and biliverdin [36, 37]. HO-1 has been reported to prevent ER stress-mediated cell apoptosis in the diabetic model [21, 26, 27]. In the present study, we discovered a previously unreported relationship between HO-1 and ER stress in the pathogenesis of ALI. Here, our results show that HO-1 could inhibit ER stress and reduce intrapulmonary cell apoptosis after sepsis through suppression of the PERK/eIF2-*α*/ATF4/CHOP pathway.

To verify our hypothesis, we first established the sepsis model and found that ALI was accompanied by ER stress 18 h after CLP surgery. Pathological score and increased water content in lungs suggested serious ALI induced by sepsis. Also, we found that the mRNA expression levels of PERK, eIF2-*α*, ATF4, and CHOP, which have been reported to be involved in one of the ER stress-mediated unfolded protein response (UPR) signaling pathways [38], were significantly increased in lung tissues of the CLP group. Furthermore, we pretreated rats with a potent ER stress inhibitor, 4-phenylbutyrate (4-PBA), before the rats were subjected to CLP surgery, and we found that 4-PBA markedly alleviated the CLP-induced ALI, reduced the upregulation of the p-PERK protein level, and downregulated the elevation of PERK, eIF2-*α*, ATF4, and CHOP mRNA levels. Our findings suggest that ER stress is one of the crucial players during CLP-induced ALI and that 4-PBA pretreatment attenuates ER stress in the pathologic condition.

The ER is an organelle that serves the role of nascent protein folding and the transportation of synthesized proteins to the Golgi body. Once the balance of ER environment is disrupted, for instance under circumstance of inflammatory stimulation or oxidative stress, a state of protein folding impairment ensues. This condition is referred to as ER stress that may initiate orchestrated UPR signaling which is aimed at restoring homeostasis of the physiological function in ER [13, 39, 40]. Otherwise, once UPR cascade is insufficient to restore the ER stress, apoptosis is initiated [13, 41, 42]. Two central components constitute UPR to sense the stress in ER [15, 17, 40]. PERK, inositol-requiring protein 1 (IRE1), and activating transcription factor-6 (ATF-6) are three specialized stress sensors located in the ER membrane. And their downstream transcription factors include eukaryotic initiation factor 2-*α* (eIF2-*α*) for PERK, fragmented ATF6 for ATF6, and spliced X-box binding protein 1 (XBP1) for IRE1. Under conditions of prolonged ER stress, UPR sensors shift their signaling to directly activate the transcription of chaperones or proteins towards inflammation or cell death [15, 38].

In diabetes-mediated endothelial apoptosis [21], the PERK is activated when being phosphorylated, then eIF-2*α* is activated to reduce the translation of general proteins. Nonetheless, the ATF-4 translation is selectively permitted for the reason that it is the prerequisite for CHOP transcription factor expression. Furthermore, CHOP is crucial in ER stress-mediated cell death by downregulating the expression of antiapoptotic proteins and upregulating the proapoptotic proteins in the Bcl-2 family [13, 43]. Consistent with other observations [13, 34], our results demonstrated that ER stress is implicated in the pathogenesis of CLP-induced pulmonary injury. In previous studies of sepsis-induced ALI, CHOP has been highlighted for it acts as an amplifier of the inflammatory response in ER stress [16, 35], but the relevant signaling pathway has not been reported. To a further extent, our results found that the PERK/eIF2-*α*/ATF4/CHOP UPR signaling contributes to the ER stress which has not been reported in the sepsis-induced ALI model.

In the present study, we also found that the HO-1 level increased after ALI. As a potent protective agent of organic injury [21, 26, 27, 36, 37], it is unclear whether or not the upregulation of HO-1 is just a simultaneous phenomenon or self-protection mechanism initiation. So, in our study, we pretreated rats which received CLP surgery with hemin (a selective HO-1 inducer) in the absence or presence of ZnPP (HO-1 inhibitor) to explore the role of HO-1 in CLP-induced lung injury. The protein level of HO-1 increased when rats was pretreated with hemin and decreased when being simultaneously treated with ZnPP. It also has been found that hemin alleviated CLP-induced ALI but ZnPP reversed the protective effects of hemin. Meanwhile, a consistent trend was found in the protein levels of p-PERK, p-eIF2*α*, ATF4, and CHOP. Furthermore, our results revealed that hemin alleviated intrapulmonary cell apoptosis, elevated the Bcl-2 level, and reduced Bax expression in lung after sepsis. ZnPP reversed the antiapoptotic effects of hemin. Thus, we speculated that HO-1 protects sepsis-induced ALI and alleviates intrapulmonary cell apoptosis through suppression of the PERK/eIF2*α*/ATF4/CHOP proapoptosis pathway in ER stress.

As a stress-inducible protein, HO-1 plays a critical role in protecting against ALI caused by pathologic variables [23, 44–47]. It has been reported that HO-1 suppressed oxidative stress in LPS-induced ALI, and the protective mechanism is associated with activation of the PI3K/Akt pathway [23] or modulation of the mitochondrial dynamic equilibrium [46]. In cigarette smoke-induced lung injury, epoxyeicosatrienoic acids (EETs) have been found to increase the expression of HO-1 and concomitantly decrease the expression of ER stress-related markers GRP78, p-eIF2-*α*, and CHOP, but the researcher did not reveal the relationship between HO-1 and ER stress [48]. To our knowledge, out results demonstrated that HO-1 exerts an antiapoptotic effect through suppression of PERK/eIF2*α*/ATF4/CHOP UPR signaling in sepsis-induced ALI for the first time.

The current study also has limitations. First of all, ATF6 and XBP1 UPR pathways also facilitate ER stress [37]. However, HO-1 has been reported to inhibit GRP78 expression which is relevant to ATF6 and XBP1 signaling [48, 49]. Second, in the context of septic ALI, some previous studies noted that HO-1-induced protection was relevant to p38 MAPK and Nrf2 signaling pathways [24, 50]. Thus, the underlying protective mechanism by which HO-1 restrains ER stress in sepsis-induced ALI is not fully expounded and requires further exploration. Third, previous studies revealed that PERK orchestrated interorganellar communication between ER and mitochondria in the context of ER stress [51, 52]. Furthermore, HO-1 has been proved modulating mitochondrial dynamic equilibrium in sepsis-induced ALI [46]. Therefore, elucidating the relationship among HO-1 expression, ER stress, and mitochondrial dynamics may have the potential value for the therapeutic target in clinical settings in future studies.

In summary, the current study demonstrated that ER stress was activated during sepsis-induced ALI. Inhibiting of ER stress could reduce ALI and improve lung function. HO-1 activation could inhibit ER stress through modulation of the PERK/eIF2-*α*/ATF4/CHOP pathway. As such, agent-facilitating HO-1 expression may be a promising strategy for preventing sepsis-induced ALI.

## Data Availability

The data used to support the findings of this study are available from the corresponding author upon request.
